# Comparing Nerve Transfer Versus Tendon Transfer and Conservative Management for Restoring Finger Extension Following Tetraplegia: A Cost-Effectiveness Analysis

**DOI:** 10.3390/jcm15207849

**Published:** 2026-10-12

**Authors:** Naveen Arunachalam Sakthiyendran, Caroline J. Cushman, Evan J. Hernandez, Brendan J. Mackay

**Affiliations:** 1 Department of Neurosurgery, Boston Medical Center, Boston University Chobanian & Avedisian School of Medicine, Boston, MA 02118, USA; 2 School of Medicine, Texas Tech University Health Sciences Center, Lubbock, TX 79430, USA; caroline.cushman@ttuhsc.edu; 3 Department of Orthopedic Surgery, University Medical Center, Texas Tech University Health Sciences Center, Lubbock, TX 79430, USA; evan.j.hernandez@ttuhsc.edu (E.J.H.);; 4 Department of Hand and Microvascular Surgery, University Medical Center, Texas Tech University Health Sciences Center, Lubbock, TX 79430, USA

**Keywords:** nerve transfer, tendon transfer, functional recovery, reconstruction, upper extremity

## Abstract

**Background/Objectives:** Cervical spinal cord injury (SCI) frequently causes tetraplegia and severe upper-limb dysfunction with substantial societal cost. Restoring finger extension is a priority, since even modest gains improve independence. Tendon transfer and supinator-to-posterior interosseous nerve transfer (SUP–PIN) both restore hand function, but their relative economic value is unclear. We assessed the lifetime cost-effectiveness of SUP–PIN versus tendon transfer and conservative management for finger extension in patients with mid-cervical SCIs who are candidates for both reconstructive strategies. **Methods:** A decision-analytic model combining a decision-tree front end with a lifetime Markov state-transition structure (one-year cycles, 3% annual discounting, half-cycle correction) compared SUP–PIN, tendon transfer, and conservative (nonoperative) care over a lifetime horizon, incorporating direct medical costs and indirect productivity losses. The outcomes were quality-adjusted life years (QALYs) accumulated over the horizon, using a willingness-to-pay threshold of $100,000/QALY. Success, complication, recovery, and return-to-work probabilities were drawn from the published literature. Deterministic, probabilistic, and two-way sensitivity analyses assessed robustness. **Results:** From the healthcare-sector perspective, conservative management was the least costly ($630.48; 14.84 QALYs) but least effective; nerve transfer was the most effective (18.55 QALYs) at $6485.84 and dominated tendon transfer, with an ICER versus conservative management of $1579/QALY, well below the $100,000/QALY threshold. From the societal perspective, nerve transfer was dominant, with the lowest lifetime cost ($903,937) and the highest effectiveness. Across the sensitivity analyses, nerve transfer was preferred under nearly all assumptions; tendon transfer was preferred only with ≥90% tendon success combined with ≤40% nerve-transfer success. **Conclusions:** In this decision-analytic model, SUP–PIN nerve transfer was projected to be economically favorable, yielding greater modeled effectiveness and lower lifetime costs than tendon transfer and conservative care under the stated assumptions. These modeled findings support further evaluation of nerve transfer in post-SCI rehabilitation algorithms and timely surgical referrals but do not, by themselves, establish clinical superiority, which requires a prospective comparative study.

## 1. Introduction

Traumatic spinal cord injury affects approximately 18,000 new patients in the U.S. annually, of whom roughly half sustain cervical-level injuries resulting in tetraplegia and severe upper limb paralysis [1]. Due to CSCI incurring significant disability and associated direct and indirect lifetime costs, estimated at $2.3–$5.1 million per patient for a cervical-level injury, the restoration of physical function remains a high priority for this population [2]. Hence, reconstructive strategies targeting these motor domains remain a top rehabilitation priority, as they offer the potential to not only improve quality of life but also reduce dependency, caregiver burden, and long-term disability costs.

To this end, a range of treatment modalities have been developed to reanimate upper limb function in patients with CSCI. Tendon-based reconstruction has historically been the standard for restoring hand function in tetraplegia and can restore finger extension either actively or passively. In an active transfer, a volitionally controlled, expendable donor muscle–tendon unit (e.g., extensor carpi radialis longus or brevis, or pronator teres) is transferred to the extensor digitorum communis to produce active digital extension; in passive tenodesis, the finger and thumb extensors (extensor digitorum communis and extensor pollicis longus) are tenodesed to the distal radius so that wrist position and gravity passively extend the digits (EDC/EPL tenodesis). Active transfers reproduce volitional digital extension but require an available expendable donor under volitional control, which is limited at mid-cervical levels; tenodesis improves controlled hand opening and release without requiring such a donor but does not reproduce independent volitional extension [3,4]. These reconstructions are reliable but, in the case of active transfers, expend a functioning donor and must be performed in advance if no target nerve is available [5]. Alternatively, nerve transfers have been adopted more recently for restoring function following tetraplegia. One particular nerve transfer, the supinator (deep branch of the radial nerve) to posterior interosseous nerve (PIN) transfer (SUP–PIN), has shown promising clinical benefit in restoring finger extension function with few complications or drawbacks [6]. Since SUP–PIN requires an intact, volitionally controlled supinator donor and a viable PIN target, it and extensor-phase tendon reconstruction are competing options specifically in patients with mid-cervical injury; the present analysis is confined to that shared candidate population.

Despite growing clinical evidence on functional outcomes, there is a lack of data on the cost-effectiveness of these reconstructive options. While prior economic analyses have demonstrated that reconstructive nerve and muscle transfers for brachial plexus or upper limb paralysis can be cost-effective, no prior study has specifically evaluated the value–proposition comparison in SCI-related treatments for finger extension [7,8,9]. To this end, we assessed the lifetime cost-effectiveness of SUP–PIN nerve transfer versus tendon transfer and conservative care for restoring finger extension post-tetraplegia. This analysis aims to inform clinicians and policymakers about the relative value of these interventions in improving patient function and reducing societal disability burden.

## 2. Materials and Methods

We developed a decision-analytic model combining a decision-tree front end with a lifetime Markov (state-transition) back end using TreeAge Pro (TreeAge Pro 2026 R2 Software, Williamstown, MA, USA) to compare three management strategies for restoring finger extension in patients with mid-cervical spinal cord injury (CSCI): (1) nerve transfer (SUP–PIN), (2) tendon transfer (e.g., ECRB/ECRL–EDC or PT–EDC), and (3) conservative management (nonoperative care including therapy and splinting). The analysis was restricted to patients with mid-cervical SCI (approximately C5–C7; motor levels in which volitional wrist extension and an expendable, volitionally controlled supinator donor are preserved) in whom restoration of finger extension is the shared reconstructive goal and for whom SUP–PIN nerve transfer and extensor-phase tendon reconstruction are both technically feasible options; patients with higher injuries lacking a suitable donor, or lower injuries with preserved digital extension, were not modeled because the two strategies are not competing alternatives in those groups. A decision tree first branched each strategy through perioperative complications to a terminal functional outcome; the resulting health state then entered a Markov structure in which the modeled cohort remained in, and accrued costs and QALYs within, that chronic health state over the patient’s remaining lifetime. A state-transition structure was chosen because the costs and health consequences of cervical SCI accrue over a patient’s remaining lifetime rather than at a single point in time; representing each year as a discrete one-year cycle allowed the cohort to accumulate costs and QALYs over the horizon. The model used a lifetime horizon to account for long-term costs and benefits, incorporating both direct medical costs and indirect productivity losses (lost work wages). The analyses were run from two perspectives so that each cost domain could be examined separately: a healthcare-sector perspective that captured only direct medical costs, and a broader societal perspective that additionally incorporated indirect productivity losses (the “income-adjusted” model). The costs and the health outcomes (measured and reported through quality-adjusted life years, QALYs) were discounted at 3% annually, and a willingness-to-pay (WTP) threshold of $100,000 per QALY was used for base-case decision-making, a standard across a majority of cost-effectiveness analysis models [7,8].

### 2.1. Model Structure and Time Horizon

The model used a one-year cycle length and a lifetime horizon. The hypothetical cohort entered at a mean age of 25 years, reflecting the peak incidence of traumatic cervical SCI, and was followed for 55 annual cycles corresponding to the base-case post-injury life expectancy of 55 years (varied 44–66 years in sensitivity analysis). During the initial cycle, each strategy determined perioperative complications (surgical arms) and final functional outcome (good recovery, MRC ≥ 3, vs. poor recovery, MRC ≤ 2); the cohort then remained in the corresponding chronic health state for the remainder of the horizon, accruing the state-specific utility and any ongoing costs each cycle. In the income-adjusted (societal) model, each recovery state was additionally divided into return-to-work and lifetime-disability substates, with annual productivity accrued accordingly. The lifetime QALYs were calculated by summing the annual health-state utility across all the cycles of the horizon, applying 3% annual discounting and a half-cycle correction; the reported effectiveness therefore represents discounted QALYs accumulated over the full lifetime horizon rather than a single cycle. The costs were accumulated and discounted on the same basis. Rather than modeling per-cycle age-specific mortality, survival was represented through the finite lifetime horizon, with varying life expectancy in the sensitivity analysis; this simplification is noted in the limitations.

The patients in each strategy had a probability of achieving a good functional outcome, defined as reaching a Medical Research Council (MRC) grade ≥ 3 on finger extension, or a poor outcome (MRC ≤ 2). The probabilities for nerve transfer/tendon transfer success and complication (e.g., wound infection, donor site morbidity) rates were derived from the previously published literature, mostly consisting of prior systematic reviews or the largest cohort studies available. Conservative management was assumed to have low likelihood of functional improvement and/or good recovery rates based on the natural history data. The costs and the health utilities were also drawn from prior published SCI studies [6,7,8,9,10,11,12,13,14,15,16,17,18,19]. Where the primary evidence for SUP–PIN pooled SCI and brachial plexus injury patients [6,14], the base-case success probability was based on the SCI/tetraplegia subset where separately reported, and the wider pooled range was used only to define the sensitivity-analysis bounds; the robustness of the conclusion to this choice is demonstrated in the one-way and two-way sensitivity analyses. Prior brachial plexus cost-effectiveness studies [7,8,9] are cited as methodological precedent for modeling nerve-transfer value, not as sources of clinical effectiveness for the present SCI population.

The cost inputs consisted of three main domains: direct treatment costs, complication management, and productivity loss. The direct costs included surgical and hospital billing estimates based on Medicare fee schedules (such as preoperative clinical and imaging/diagnostic studies, anesthesia, surgeon fees, and implants). The complication costs were estimated using national data from the Healthcare Cost and Utilization Project (HCUP) and Medicare claims databases, reflecting expenses related to managing commonly encountered postoperative issues in nerve and tendon transfers. The productivity loss was modeled as the lifetime income differential between patients returning to work versus those remaining unemployed (due to disability), calculated using national median wage data from the U.S. Bureau of Labor Statistics. All the base-case parameter values, ranges, distributions, and sources are summarized in Table 1, and the individual cost components with their corresponding CPT codes are itemized in Table A1. All the cost inputs are reported in 2024 U.S. dollars. The direct surgical and clinical costs were taken from the 2024 Medicare Physician Fee Schedule; the complication-management costs from the most recent available HCUP/Medicare data; and the wage data from the 2023 U.S. Bureau of Labor Statistics median annual wage. Where the source data predated 2024, the values were inflation-adjusted to 2024 dollars using the medical-care component of the Consumer Price Index for healthcare costs and the all-items CPI for wages.

### 2.2. Building the Model

**Figure 1:**
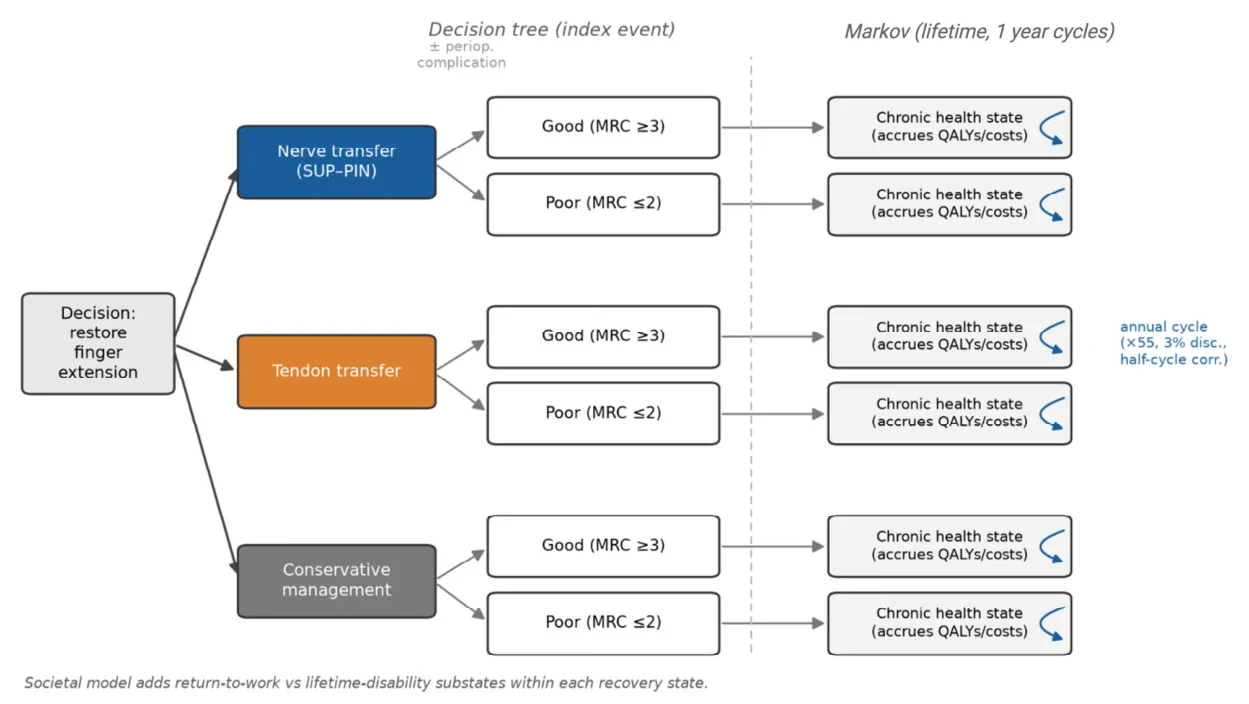
Structure of the decision-analytic model. The model combines a decision-tree front end with a lifetime Markov state-transition back end, comparing three strategies for restoring finger extension after cervical spinal cord injury: supinator-to-posterior interosseous nerve (SUP–PIN) transfer, tendon transfer, and conservative (nonoperative) management. In the decision-tree component, each surgical arm first branches on the probability of perioperative complications and then reaches a “good” (Medical Research Council [MRC] grade ≥ 3) or “poor” (MRC grade ≤ 2) functional outcome; conservative management branches directly to a good or poor outcome. In the Markov component, the resulting outcome defines a chronic health state that the cohort occupies over the lifetime horizon, cycling annually (one-year cycles) and accruing state-specific costs and QALYs until the end of the horizon.

The model was built as follows: (1) surgical strategies (nerve and tendon transfer) first branched through the (2) probability of perioperative complications, each leading to final functional outcomes: (3) “good” motor recovery (MRC ≥ 3) or “poor” recovery (MRC ≤ 2). The conservative management node directly led to good or poor outcomes/recovery. In the model with income adjustment, return-to-work rates vs. lifetime-disability rates were modeled as the terminal branch for each node. The return-to-work rate was conditional on motor recovery. This branch order mirrors the clinical sequence, in which perioperative complications arise before a final functional state is reached, and making return to work conditional on motor recovery reflects that the ability to resume employment depends on the degree of hand function regained. The non-income-adjusted and income-adjusted models are illustrated in Figure 1 for reference.

### 2.3. Statistical Analysis

A base-case analysis was conducted to evaluate the lifetime cost-effectiveness of nerve transfer, tendon transfer, and conservative management, both with and without income adjustment. For each strategy, total costs, utilities/quality-adjusted life years (QALYs), incremental cost-effectiveness ratios (ICERs), and net monetary benefit (NMB) were calculated using a willingness-to-pay (WTP) threshold of $100,000/QALY.

To assess parameter uncertainty and identify influential inputs, one-way deterministic sensitivity analyses were performed on all the major variables in the income-adjusted model, including probabilities of achieving good motor recovery (MRC ≥ 3), utility values for different health states, treatment costs, and probabilities of returning to work following good or poor outcomes. Each variable was independently varied across plausible ranges to determine its impact on the model’s ICERs and NMB. The nerve and tendon transfer outputs for the one-way sensitivity analysis were compared against conservative management for all the variations.

Two-way sensitivity analyses were also conducted to explore the interactive effects of the key clinical parameters. Specifically, joint variations in the probabilities of good outcomes for nerve transfer versus tendon transfer in both the non-income-adjusted and income-adjusted models were evaluated to identify the thresholds at which the preferred strategy changed.

To account for uncertainty across all the parameters simultaneously, a probabilistic sensitivity analysis (PSA) was performed using 100,000 Monte Carlo simulations, each of which drew a random value for every parameter from its assigned distribution so that all input uncertainty was varied simultaneously; this large number of iterations was used to obtain stable estimates of the probability that each strategy was cost-effective. Beta distributions were used for probabilities and utilities, and gamma distributions for cost parameters. The resulting outputs were used to construct cost-effectiveness acceptability curves (CEACs) across a WTP range of $0 to $100,000/QALY, illustrating the likelihood of each strategy being optimal at varying WTP thresholds. A half-cycle correction was applied throughout the Markov model to reflect mid-cycle transitions.

## 3. Results

### 3.1. Base Case (Without Income Adjustment)

In the base-case analysis from the healthcare-sector perspective (without income adjustment), conservative management was the least costly ($630.48) but yielded the lowest effectiveness (14.84 QALYs) and an NMB of $1,483,744 at a willingness-to-pay (WTP) of $100,000/QALY. Nerve transfer provided the highest effectiveness (18.55 QALYs) at a cost of $6485.84, with an incremental cost of $5855.36 and an incremental gain of 3.71 QALYs versus conservative management, yielding an ICER of $1579/QALY and the highest NMB ($1,848,760). Tendon transfer cost $6682.60 and produced 18.32 QALYs; since it was both more costly and less effective than nerve transfer, it was dominated by nerve transfer (ICER versus conservative management, $1742/QALY; NMB $1,825,122). Effectiveness is reported as QALYs accumulated and discounted over the lifetime horizon.

**Figure 2:**
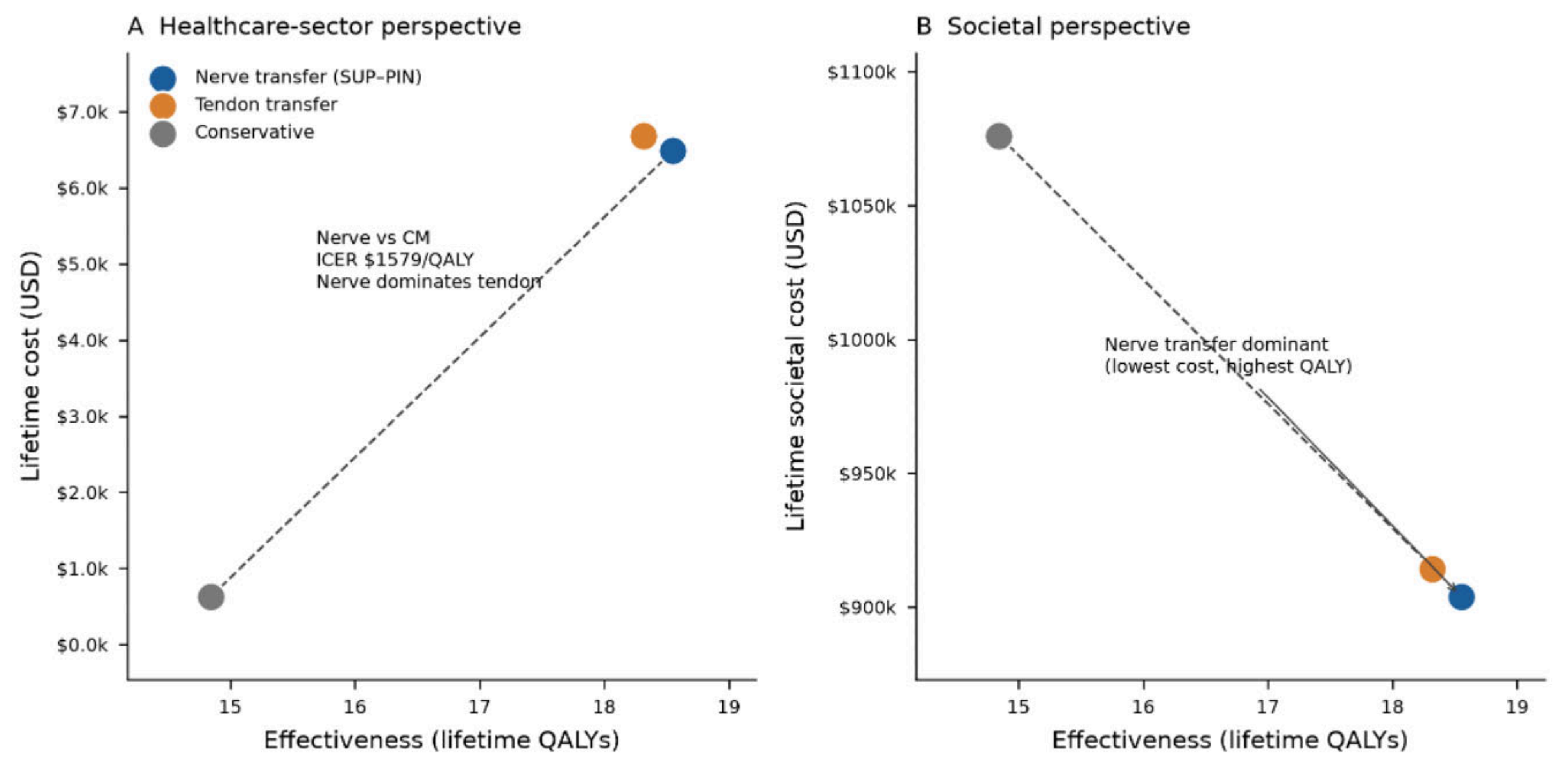
Base-case cost-effectiveness results. Lifetime costs and effectiveness (quality-adjusted life years, QALYs accumulated and discounted over the lifetime horizon) for nerve transfer, tendon transfer, and conservative management, evaluated at a willingness-to-pay (WTP) threshold of $100,000/QALY. (**A**) Healthcare-sector perspective (direct medical costs only): nerve transfer produced the highest net monetary benefit and dominated tendon transfer (ICER vs. conservative management, $1579/QALY). (**B**) Societal perspective (direct medical costs plus productivity losses): nerve transfer was dominant, with the lowest lifetime cost and highest effectiveness. The dashed line connects conservative management to nerve transfer on the cost-effectiveness plane.

From the societal perspective (with productivity loss included and future wages discounted at 3% annually), nerve transfer was both the least costly and the most effective strategy. Nerve transfer yielded the lowest total lifetime cost ($903,936.98) and the highest effectiveness (18.55 QALYs), with an NMB of $951,309. Tendon transfer cost $914,335.84 (18.32 QALYs; NMB $917,469), and conservative management was the most costly ($1,076,051.54) and the least effective (14.84 QALYs; NMB $408,323). Since nerve transfer was less costly and more effective than both comparators, it was the dominant strategy from the societal perspective. The results from the base-case analysis are illustrated in Figure 2.

### 3.2. Tornado Sensitivity Analysis (Income-Adjusted)

**Figure 3:**
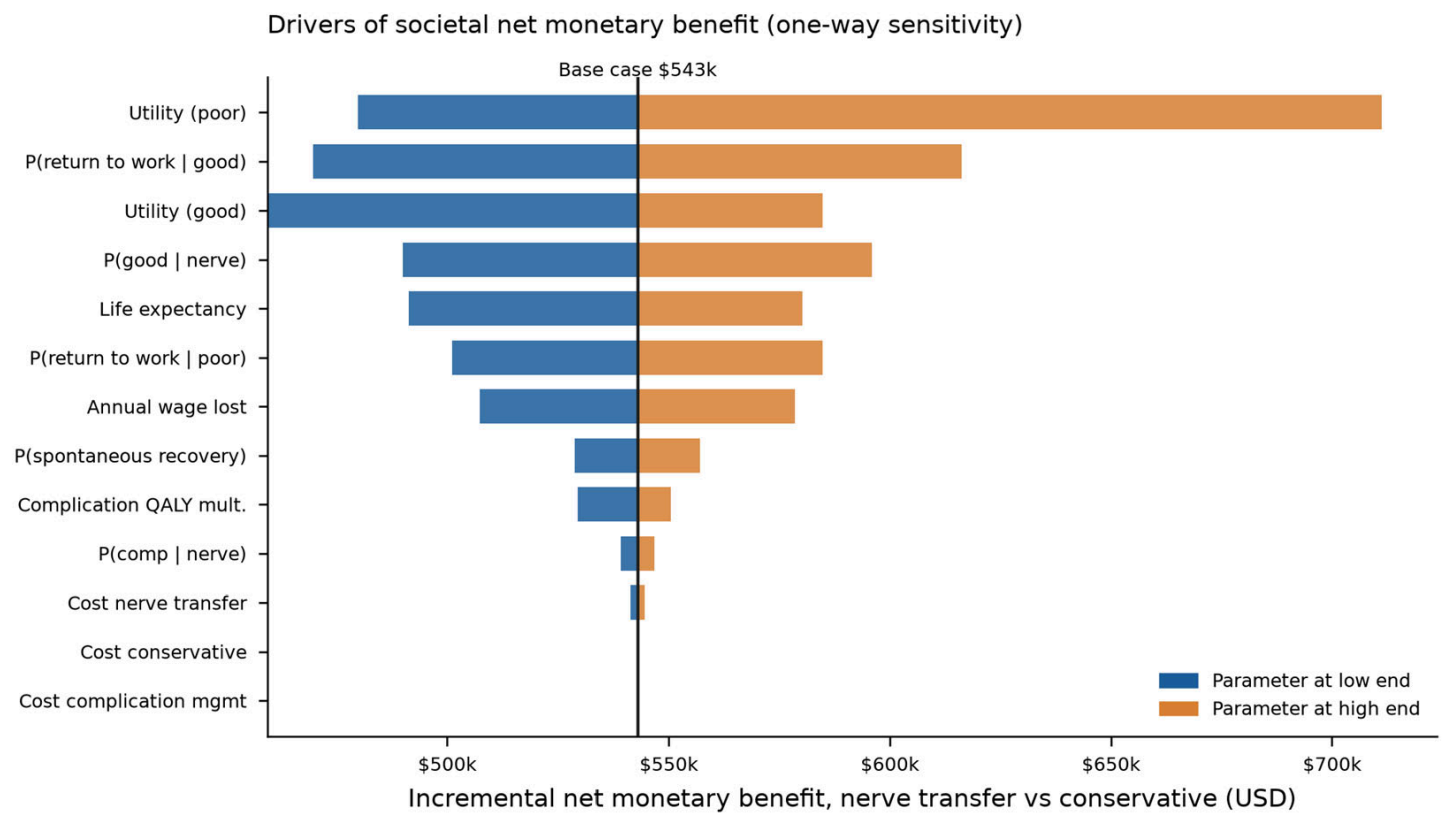
One-way (deterministic) sensitivity analysis. Tornado diagram ranking model inputs by their influence on the incremental net monetary benefit (NMB) of nerve transfer versus conservative management in the societal (income-adjusted) model at a WTP of $100,000/QALY; each bar shows the range of incremental NMB produced as the corresponding parameter was varied across its plausible range with all others held at base-case values. The vertical line marks the base-case incremental NMB. The incremental NMB remained positive across the full range of every parameter, so nerve transfer was favored throughout.

In the societal (income-adjusted) model, the incremental net monetary benefit of nerve transfer versus conservative management was most sensitive to the health-state utility for poor recovery, followed by the probability of returning to work after good recovery and the utility for good recovery; the probability of a good outcome following nerve transfer and life expectancy were also influential, whereas the surgical cost and the complication rates had minimal impact (Figure 3). Across the full plausible range of every parameter, the incremental net monetary benefit of nerve transfer remained strongly positive, so nerve transfer was favored throughout.

### 3.3. One-Way Sensitivity Analysis

From the healthcare-sector perspective, the ICER for nerve transfer versus conservative management was most sensitive to the health-state utilities and to the probability of a good outcome following nerve transfer but remained far below the $100,000/QALY threshold throughout. As the probability of a good outcome following nerve transfer varied from 79% to 94%, the ICER ranged from $1749 to $1439/QALY. Across the same range of nerve-transfer success, tendon transfer remained dominated by nerve transfer in every scenario. When the tendon-transfer success was varied from 70% to 95%, the tendon ICER versus conservative management ranged from $2088 to $1477/QALY, remaining higher than that of nerve transfer except at the highest tendon-success/lowest nerve-success combinations.

Varying the health-state utilities had the largest influence on the ICER. As the utility of a good recovery increased from 0.68 to 0.74, the ICER for nerve transfer versus conservative management decreased from $2039 to $1419/QALY, and that for tendon transfer from $2253 to $1565/QALY. As the utility for poor recovery increased from 0.46 to 0.57, the ICER for nerve transfer increased from $1086 to $1902/QALY, and that for tendon transfer from $1196 to $2102/QALY. In every one-way scenario, nerve transfer remained cost-effective at the $100,000/QALY threshold and dominated tendon transfer.

From the societal perspective, nerve transfer remained the dominant strategy (less costly and more effective than both comparators) across the full plausible range of every parameter tested, including the probability of a good outcome following nerve transfer (79–94%) and the probabilities of returning to work following a good (20–50%) or poor (10–30%) recovery. The results of the one-way sensitivity analysis are detailed in Table 2.

### 3.4. Two-Way Sensitivity Analysis—Non-Income-Adjusted

From the healthcare-sector perspective, nerve transfer produced the highest net monetary benefit (NMB) whenever its probability of a good outcome was ≥50–60% and tendon transfer remained ≤70%. For instance, at a 70% success probability for nerve transfer and 30% for tendon transfer, nerve transfer yielded an NMB of $1,769,558 compared with $1,483,744 for conservative management and $1,575,766 for tendon transfer.

Tendon transfer became the preferred option only when its success rate reached ≥90% and nerve transfer dropped to ≤40%. For example, at 90% tendon and 30% nerve success probabilities, tendon transfer achieved an NMB of $1,863,484 versus $1,577,554 for nerve transfer and $1,483,744 for conservative management.

Across all the modeled combinations, conservative management yielded the lowest NMB whenever either surgical strategy achieved a moderate success probability, fixed at $1,483,744. In direct comparisons, nerve transfer dominated conservative management across essentially the entire plausible range of nerve-transfer success; only at low nerve-transfer success probabilities combined with low utility did the margin narrow.

From the societal perspective, nerve transfer yielded the highest NMB whenever its probability of a good outcome was ≥50% and tendon transfer remained ≤70%. For example, at a 60% success probability for nerve transfer and 30% for tendon transfer, nerve transfer achieved an NMB of $764,027, outperforming both tendon transfer ($550,222) and conservative management ($408,323).

Tendon transfer became preferred only when its probability of success was ≥90% and nerve transfer was ≤40%. Under these conditions, tendon transfer achieved an NMB of $973,968 (at 90% tendon, 30% nerve success), outperforming nerve transfer ($552,009) and conservative management ($408,323).

**Figure 4:**
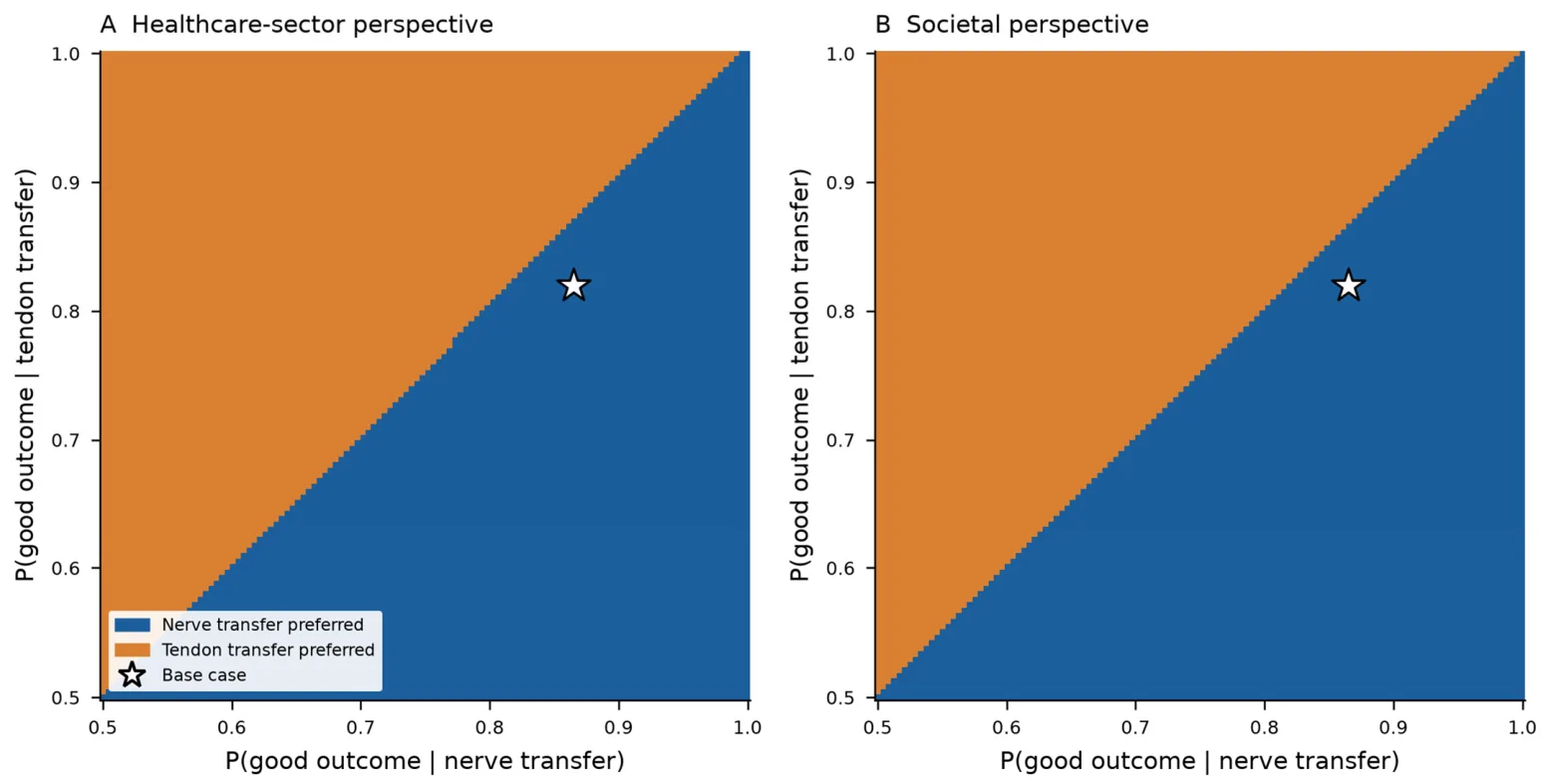
Two-way sensitivity analysis. Preferred (highest-NMB) strategy across simultaneous variation in the probability of a good outcome following nerve transfer and following tendon transfer, from both the healthcare-sector and societal perspectives, using lifetime-accumulated QALYs. The star marks the base case. Nerve transfer yielded the highest NMB across most of the parameter space; tendon transfer became preferred only when its success probability was ≥90% and nerve-transfer success fell to ≤40%.

Conservative management remained the least favorable strategy in nearly all the scenarios, with an NMB near $408,323. When comparing nerve transfer to conservative management, nerve transfer dominated across the full plausible range of success probabilities. The results from the two-way sensitivity analysis are illustrated in Figure 4.

### 3.5. Probabilistic Sensitivity Analysis

The CEACs showed that from the societal perspective, nerve transfer had the highest acceptability (62.1–64.0%) across all the willingness-to-pay thresholds (range $0–$100,000/QALY). Tendon transfer was acceptable in approximately 36.4% of the iterations, while conservative management remained below 1.8%.

**Figure 5:**
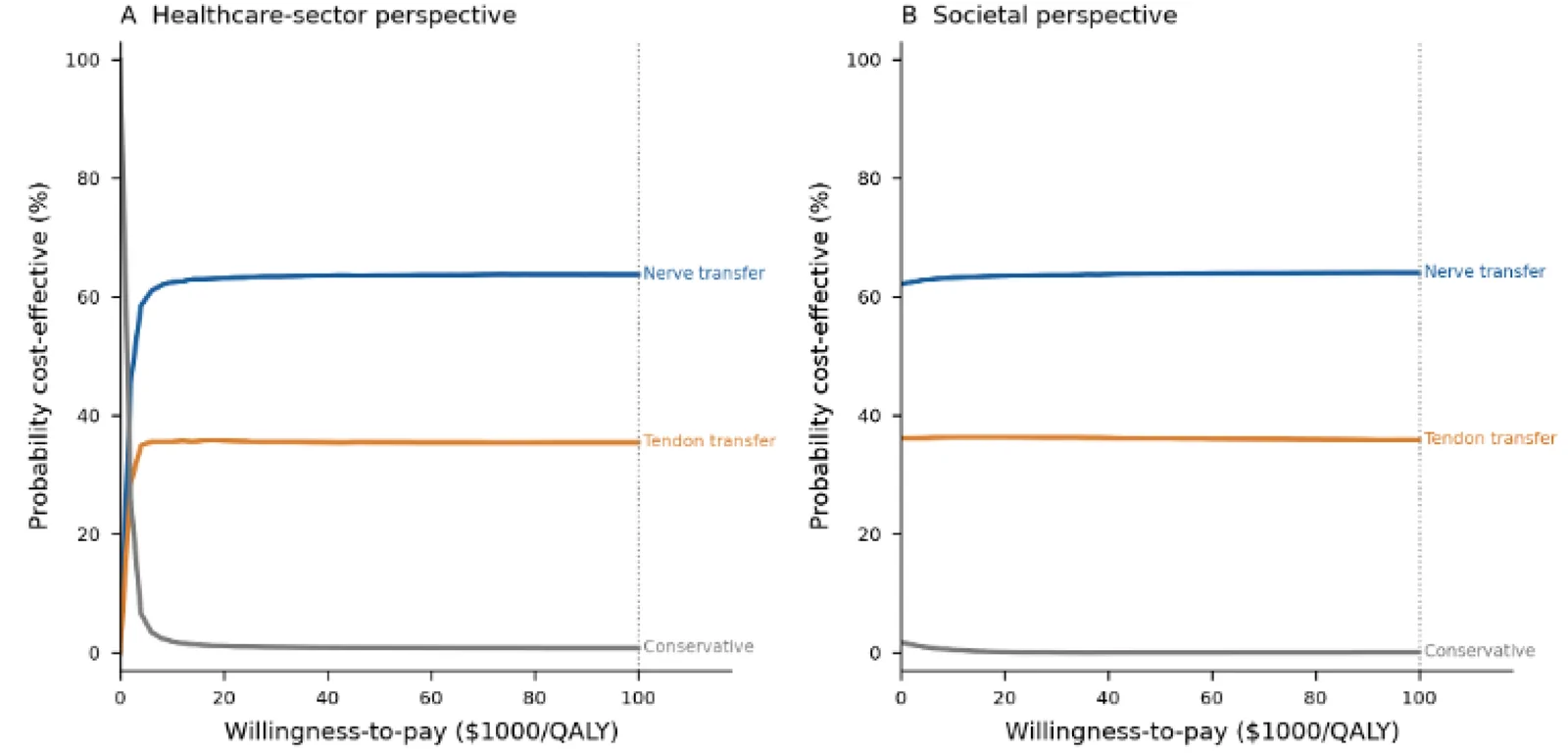
Cost-effectiveness acceptability curves (CEACs). Probability that each strategy is the most cost-effective option across WTP thresholds from $0 to $100,000/QALY, from a probabilistic sensitivity analysis of 20,000 Monte Carlo simulations on lifetime-accumulated QALYs. From the societal perspective, nerve transfer had the highest probability of being cost-effective (64.0% at $100,000/QALY) across thresholds; from the healthcare-sector perspective, conservative management was most likely to be cost-effective only at very low thresholds, while nerve transfer became the most probable above approximately $2000/QALY (63.7% at $100,000/QALY).

From the healthcare-sector perspective, conservative management had the highest probability of being cost-effective only at very low WTP thresholds (100% at WTP = $0), declining to 0.8% at $100,000/QALY. Nerve transfer increased from 0% to 63.7%, becoming the option with the highest probability of being cost-effective above approximately $2000/QALY. Tendon transfer increased from 0% to 35.4% but never surpassed nerve transfer. From the societal perspective, nerve transfer had the highest probability of being cost-effective (64.0% at $100,000/QALY) across the full range of thresholds. The CEACs are illustrated in Figure 5.

## 4. Discussion

In this health economics analysis comparing the costs and utilities of different strategies, the SUP–PIN nerve transfer delivered the highest modeled value for restoring meaningful upper-limb finger extension function following CSCI in anatomically eligible candidates. Specifically, when accounting for both healthcare costs and lifetime productivity losses, nerve transfer yielded the greatest effectiveness at the lowest total cost, resulting in the highest net monetary benefit. By comparison, tendon transfer was slightly more expensive and modestly less effective, and conservative management, while the cheapest upfront, led to the poorest modeled outcomes and the highest lifetime societal cost.

Nerve transfer offers distinct mechanistic, functional, and practical advantages over tendon transfer for restoring finger extension in the SCI patient population. Surgically, nerve transfers involve coapting a viable, redundant, and expendable donor nerve with an intact upper motor input to the motor branch of a non-functional target [6,20]. This facilitates the reinnervation of native muscles, enabling active, volitional extension across multiple digits through a single motor command [20,21]. Tendon transfers, by contrast, restore digital extension by mechanically rerouting a functioning donor tendon to the paralyzed extensors (active transfer) or by tenodesis to the radius (passive hand opening); in either case, the reconstruction expends a donor’s original function, whereas nerve transfer preserves joint biomechanics, avoids sacrificing essential donor functions, and restores more physiologically natural movement [5,22]. Active tendon transfers can reproduce volitional digital extension but depend on precise tensioning to balance extension across all the digits and, similar to tenodesis, may degrade over time due to elongation of the transferred muscle or adhesions with loss of tendon excursion [22]. In contrast, when performed within the optimal time window, nerve transfers reestablish active, coordinated movement through biologic reinnervation, offering durable control without the risks outlined above, not typically achievable through mechanical rerouting alone [6].

The findings from our analysis also highlight the critical clinical and economic importance of restoring active finger extension in CSCIs. While the modeled cost savings were partially attributable to improved return-to-work rates, the more universal and substantial benefits lie in enabling the foundational activities of daily living (such as self-feeding, grooming, hygiene, and toileting) that restore autonomy, reduce caregiver dependence, and elevate quality of life [23]. For the vast majority of individuals with mid-cervical injuries, these gains represent the critical impact of upper limb reanimation. In this context, the utility benefit and return-to-work rates indicated in our model operate as a surrogate marker for broader gains in functional health, reflecting improvements in autonomy, caregiver reliance, and societal reintegration.

Despite the clinical and economic efficacy, nerve transfers remain notably underutilized in the contemporary management of tetraplegia. The contributing factors include the evolving and more recent integration of this strategy in the context of tetraplegia, delayed referral timelines, insufficient provider awareness, and inconsistent integration into rehabilitation frameworks [24]. Within the assumptions of our model, nerve transfer was the economically preferred strategy relative to tendon transfer across virtually all the evaluated scenarios, with the sensitivity analyses supporting this preference even at modest success/good-outcome rates. These modeled results, together with the finite window during which reinnervation remains feasible, support early evaluation and intervention [13]. When anatomically feasible, SUP–PIN nerve transfer may be a preferred first-line option for restoring finger extension in suitable candidates, pending prospective confirmation. Tendon transfer, although valuable in select cases with chronic denervation, older populations, or limited donor nerves, remains an important option in anatomically constrained scenarios. From a systems and policy perspective, these modeled data support further consideration of nerve transfer within routine post-SCI treatment algorithms and its recognition as a high-value surgical intervention. Promoting early surgical consultation, expanding provider education, and incorporating cost-effectiveness evidence into clinical guidelines may help ensure timely access to nerve reconstruction, with the goal of maximizing patient outcomes while minimizing long-term societal costs [13].

Our model, similar to any decision-analytic tool, relies on averaged data from the literature and cannot capture every unique clinical scenario or patient-specific variability. Factors such as individual comorbidities, exact injury levels, or psychosocial elements may influence the real-world outcomes beyond what the model takes into consideration. Several specific limitations warrant emphasis. First, the comparison is confined to the mid-cervical subgroup eligible for both SUP–PIN and tendon-based finger-extension reconstruction; it does not address patients with higher or lower injuries, nor the full menu of tendon-transfer procedures used across different motor levels, so the conclusions apply to the specific decision of how best to restore finger extension in an eligible candidate rather than to reconstruction across all tetraplegia levels. Second, this is an indirect comparison; the success probabilities, utilities, and complication rates were drawn from separate single-arm cohorts and systematic reviews rather than from a head-to-head trial, and part of the SUP–PIN effectiveness evidence derives from cohorts that combined spinal cord and brachial plexus injury, reflecting the limited SCI-only literature; residual confounding between evidence sources therefore cannot be excluded, although our sensitivity analyses were designed to bracket this uncertainty and the preferred strategy did not change across the plausible range. Third, the return-to-work and productivity assumptions carry substantial uncertainty and strongly influence the societal-perspective results—the tornado analysis identified the probability of returning to work after a good recovery as the single most influential parameter (Figure 3)—so these assumptions should be regarded as a key driver of, and a source of uncertainty in, the societal findings. Indeed, the clinical reality of return to work may never be an appreciably achievable goal for the majority of patients with tetraplegia under the current standards of care; the income-adjusted analysis, while methodologically appropriate from a societal perspective, likely overestimates the productivity gains by extrapolating employment potential that is not representative of typical CSCI outcomes. Finally, survival was represented through a finite lifetime horizon with varying life expectancy in the sensitivity analysis rather than through per-cycle age-specific mortality transitions, a simplification that may not fully capture differential survival across the health states.

An additional limitation concerns the definition of a “good” functional outcome. We dichotomized recovery at an MRC grade ≥ 3; however, an MRC grade of 3—active finger extension against gravity but not against resistance—does not necessarily confer the robust, task-ready hand function that patients and clinicians would regard as an excellent result, and it has been argued to represent a comparatively modest endpoint. This threshold was nonetheless adopted because the largest cohort studies and systematic reviews of nerve and tendon transfer in tetraplegia most consistently report motor outcomes dichotomized at MRC ≥ 3, which maximized the quantity and comparability of the source data available for parameterizing the model. A stricter cutoff (e.g., MRC ≥ 4) would more closely capture high-functioning recovery but would have markedly reduced the available evidence base. Hence, as more granular outcome data emerge, future models may be able to incorporate functionally graded recovery states rather than a single dichotomous threshold.

## 5. Conclusions

Within the assumptions of this decision-analytic model, SUP–PIN nerve transfer was the most cost-effective strategy for restoring finger extension in eligible patients with mid-cervical SCI, projected to provide greater modeled effectiveness and lower lifetime costs than tendon transfer and conservative management. These modeled findings support further evaluation and, where anatomically appropriate, a consideration of nerve transfer in post-SCI rehabilitation algorithms; they should be confirmed by prospective comparative studies before its superiority can be regarded as established. Future research should validate these results through prospective clinical trials and incorporate broader societal perspectives to further refine decision-making in SCI rehabilitation.

## Figures and Tables

**Table 1:** Base case values/parameters.

Model Parameters	Mean, Base Case	SD (for PSA)	Range (for DSA)	Distribution Type	Source
Annual Wage Lost in Disability	49,809	9962	39,847–59,771	Gamma	30371909
Bad Utility	0.54	0.05	0.46–0.57	Beta	16084817
Good Utility	0.72	0.05	0.68–0.74	Beta	19896010, 16084817
Complications QALY Multiplier	0.90	0.18	0.72–1	Beta	30371909
Conservative Management Costs	630.48	134.07	536.30–804.44	Gamma	CDC Medicare Reimbursement Website
Spontaneous Recovery Probability	0.08	0.02	0.06–0.10	Beta	8439249, 35896613
Cost of Complication Management	1376	516.6	1032.00–2064.00	Gamma	CDC Medicare Reimbursement Website
Probability of Complications after Nerve Transfer	0.04	0.01	0.02–0.04	Beta	38224237
Cost of Nerve Transfer	6430.8	1045.96	4825.00–8036.00	Gamma	CDC Medicare Reimbursement Website
Probability of Good Outcomes after Nerve Transfer	0.865	0.17	0.79–0.94	Beta	38224237, 39094196, 36441549
Probability of Complications after Tendon Transfer	0.05	0.01	0.04–0.06	Beta	27346724, 27233586
Cost of Tendon Transfer	6613.80	1322.76	5291.04–7936.56	Gamma	
Probability of Good Outcomes after Tendon Transfer	0.82	0.16	0.66–0.98	Beta	22144751, 16928411
Life Expectancy (after 25)	55	11	44–66	Normal	Standard
Probability of Returning to Work after Good Recovery	0.35	0.07	0.28–0.42	Beta	36717734
Probability of Returning to Work after Poor Recovery	0.18	0.04	0.14–0.22	20025147Beta	20025147
Discount Rate	0.03				Standard

The SD and Distribution Type columns parameterize the probabilistic sensitivity analysis (PSA; beta distributions for probabilities and utilities, gamma for costs). The Range column gives the bounds used for the one-way deterministic sensitivity analyses (DSAs). For nerve-transfer good-outcome probability, the base case is 0.865 (86.5%) with a DSA range of 0.79–0.94; the wider 0.70–1.00 interval reported by the pooled brachial plexus/SCI literature was used only to set the PSA SD. Numeric source identifiers are PubMed IDs.

**Table 2:** One-way sensitivity analysis results.

Variable	Variable Value	Strategy	ICER vs. CM
Probability of Good Outcomes (MRC ≥ 3) Following Nerve Transfer	79%	Nerve Transfer	$1749
		Tendon Transfer	$1742
	82%	Nerve Transfer	$1676
		Tendon Transfer	$1742
	86.5% (base)	Nerve Transfer	$1579
		Tendon Transfer	$1742
	90%	Nerve Transfer	$1510
		Tendon Transfer	$1742
	94%	Nerve Transfer	$1439
		Tendon Transfer	$1742
Probability of Good Outcomes (MRC ≥ 3) Following Tendon Transfer	70%	Nerve Transfer	$1579
		Tendon Transfer	$2088
	75%	Nerve Transfer	$1579
		Tendon Transfer	$1928
	82% (base)	Nerve Transfer	$1579
		Tendon Transfer	$1742
	90%	Nerve Transfer	$1579
		Tendon Transfer	$1569
	95%	Nerve Transfer	$1579
		Tendon Transfer	$1477
Utility, Good Recovery	0.68	Nerve Transfer	$2039
		Tendon Transfer	$2253
	0.70	Nerve Transfer	$1780
		Tendon Transfer	$1965
	0.72 (base)	Nerve Transfer	$1579
		Tendon Transfer	$1742
	0.74	Nerve Transfer	$1419
		Tendon Transfer	$1565
Utility, Poor Recovery	0.46	Nerve Transfer	$1086
		Tendon Transfer	$1196
	0.50	Nerve Transfer	$1287
		Tendon Transfer	$1418
	0.54 (base)	Nerve Transfer	$1579
		Tendon Transfer	$1742
	0.57	Nerve Transfer	$1902
		Tendon Transfer	$2102

## Data Availability

The supplementary spreadsheet containing all model inputs, probability distributions, cost components, and effectiveness calculations are available from the corresponding author on reasonable request.

## Abbreviations

The following abbreviations are used in this manuscript:

CEAC(s)	Cost-Effectiveness Acceptability Curve(s)
CPT	Current Procedural Terminology
CSCI	Cervical Spinal Cord Injury
ECRB	Extensor Carpi Radialis Brevis
ECRL	Extensor Carpi Radialis Longus
EDC	Extensor Digitorum Communis
EPL	Extensor Pollicis Longus
HCUP	Healthcare Cost and Utilization Project
ICER(s)	Incremental Cost-Effectiveness Ratio(s)
MRC	Medical Research Council
NMB	Net Monetary Benefit
PIN	Posterior Interosseous Nerve
PSA	Probabilistic Sensitivity Analysis
PT	Pronator Teres
QALY(s)	Quality-Adjusted Life Year(s)
SCI	Spinal Cord Injury
SUP–PIN	Supinator-to-Posterior Interosseous Nerve (Transfer)
WTP	Willingness-to-Pay

## Appendix A

**Table A1:** Cost component table.

Cost Component	Cost (Fees)	CPT Code
Nerve transfer surgery + facility fee	$4062	64905
Tendon transfer surgery + facility fee	$4245	27691
Anesthesia	$670.00	01710
New clinic visit	$207.29	99205
Pre- & postop surgical visits	$976.32	99203
EMG conduction study	$197.62	95910
Preop EMG	$92.00	95886
Preop cervical spine MRI	$225.57	72141
